# Increased accuracy in identifying NAFLD with advanced fibrosis and cirrhosis: independent validation of the Agile 3+ and 4 scores

**DOI:** 10.1097/HC9.0000000000000055

**Published:** 2023-05-04

**Authors:** Mazen Noureddin, Edward Mena, Raj Vuppalanchi, Niharika Samala, Micaela Wong, Fabiana Pacheco, Prido Polanco, Celine Sakkal, Ani Antaramian, Devon Chang, Nabil Noureddin, Anita Kohli, Stephen A. Harrison, Samer Gwarieh, Naim Alkhouri, Emily Truong

**Affiliations:** 1 Houston Methodist Hospital, Houston Research Institute, Houston, Texas, USA; 2 California Liver Institute, Pasadena, California, USA; 3 Indiana University, Indianapolis, Indiana, USA; 4 Arizona Liver Health, Phoenix, Arizona, USA; 5 Comprehensive Transplant Center, Los Angeles, California, USA; 6 Cedars-Sinai Medical Center, Los Angeles, California, USA; 7 Arnold O. Beckman High School, Irvine, California, USA; 8 Division of Gastroenterology, University of California at San Diego, La Jolla, California, USA; 9 Radcliffe Department of Medicine, University of Oxford, England, UK; 10 Department of Medicine Center, Los Angeles, California, USA; 11 Cedars-Sinai Medical Center, Los Angeles, California, USA

## Abstract

We explored 2 novel scores, Agile 3+ and 4, to identify advanced fibrosis (≥F3) and cirrhosis (F4), respectively, in NAFLD and compared their diagnostic performances to liver stiffness measurement (LSM) by vibration-controlled transient elastography and fibrosis-4 index (FIB-4) (for Agile 3+).

**Approach and Results:**

This multicenter study included 548 NAFLD patients with laboratory testing, liver biopsy, and vibration-controlled transient elastography within 6 months. Agile 3+ and 4 were applied and compared with FIB-4 or LSM alone. Goodness of fit was evaluated using a calibration plot and discrimination using area under the receiver operating curve. Area under the receiver operating curves was compared using the Delong test. Dual cutoff approaches were applied to rule out and rule in ≥F3 and F4. Median (interquartile range) age was 58 (15) years. Median body mass index was 33.3 (8.5) kg/m^2^. Fifty-three percent had type 2 diabetes, 20% had F3, and 26% had F4. Agile 3+ demonstrated an area under the receiver operating curve of 0.85 (0.81; 0.88) similar to that of LSM [0.83 (0.79; 0.86), *p*=0.142] but significantly higher than that of FIB-4 [0.77 (0.73; 0.81), *p*<0.0001). Agile 4’s area under the receiver operating curve [0.85 (0.81; 0.88)] was similar to that of LSM [0.85 (0.81; 0.88), *p*=0.065). However, the percentage of patients with indeterminate results was significantly lower with Agile scores compared with FIB-4 and LSM (Agile 3+: 14% vs. FIB-4: 31% vs. LSM: 13%, *p*<0.001; Agile 4: 23% vs. LSM: 38%, *p*<0.001).

**Conclusions:**

Agile 3+ and 4 are novel vibration-controlled transient elastography–based noninvasive scores that increase accuracy in the identification of advanced fibrosis and cirrhosis respectively and are ideal for clinical use due to a lower percentage of indeterminant outputs compared with FIB-4 or LSM alone.

## Graphical abstract

**Figure:**
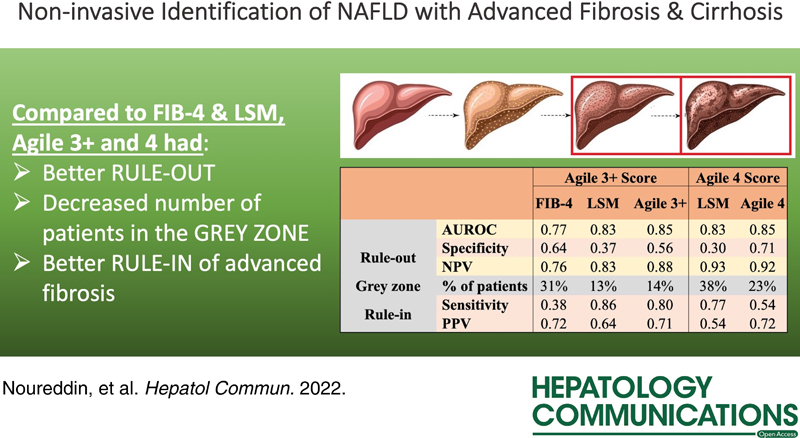

NAFLD is the most common cause of chronic liver disease worldwide, affecting 25%–35% of the global adult population and up to 70% of those with type 2 diabetes and obesity.^1^ Because NASH is associated with fibrosis, cirrhosis, and even HCC, NAFLD is one of the leading indicators for liver transplantation in Europe and the US.^1^,^2^ As NAFLD is often clinically silent, a key challenge is identifying those with advanced fibrosis (fibrosis stage of ≥F3) and cirrhosis (F4) who are at significantly higher risk of liver-related mortality.^3^

Currently, risk stratification of liver fibrosis ranges from noninvasive assessment scores to percutaneous liver biopsy. Although the reference method to assess liver fibrosis, liver biopsy is invasive and limited by cost, sampling variability, and intrareader/interreader variability.^4^ Noninvasive modalities to risk stratify fibrosis include serum biomarkers, imaging, and algorithms combining both. Among the serum biomarkers, fibrosis-4 index (FIB-4) and NAFLD fibrosis score have demonstrated high accuracy in excluding advanced fibrosis with negative predictive values >90%.^5^ In regard to imaging modalities, liver stiffness measurement (LSM) by vibration-controlled transient elastography (VCTE) is accurate in excluding advanced fibrosis and cirrhosis with negative predictive values of ~90%.^6^ However, FIB-4, NAFLD fibrosis score, and LSM by VCTE are inadequate for ruling in advanced fibrosis or cirrhosis and often necessitate additional testing in the case of positive results.^7^ The novel Fibroscan-based Agile 3+ and 4 scores combining LSM by VCTE with constitutive demographic data (age, sex, and presence of type 2 diabetes) and serum biomarkers (aspartate aminotransferase, alanine transaminase, and platelets) were recently introduced to better rule in advanced fibrosis and cirrhosis, respectively, in NAFLD.^8^,^9^

We aimed to perform an independent validation of the Agile 3+ and 4 scores performance for diagnosing advanced fibrosis and cirrhosis, respectively, in a mulicenter real-world cohort of NAFLD patients in the US. We further compared the Agile 3+ and 4 score performances to FIB-4 and LSM using previously published cutoff values.

## PATIENTS AND METHODS

### Participants

This analysis included adults with biopsy-proven NAFLD from 1 outpatient clinic and 3 tertiary care centers from Arizona (Arizona Liver Health), California (Cedars Sinai Medical Center, California Liver Institute), and Indiana (Indiana University) in the US.

Data were collected between 2014 and 2021 from patients with NAFLD who underwent liver biopsy, VCTE, and laboratory tests within 6 months. Subjects were excluded for the following: (1) missing data necessary for calculating the Agile 3+ and 4 scores, (2) missing fibrosis stage on liver biopsy, or (3) history of chronic liver disease other than NAFLD including autoimmune hepatitis, α1-antitrypsin deficiency, chronic hepatitis B or C, primary sclerosing cholangitis, primary biliary cholangitis, hemochromatosis, Wilson disease, or medications that can drive hepatic steatosis. Patients completed in-depth medical history, physical examination, and laboratory assessment before undergoing LSM. Liver biopsy was performed in the presence of abnormalities that raised concern for clinically significant NAFLD such as high LSM or diabetes, with or without elevated liver biochemistries. This study was exempt from institutional review board.

### Vibration-controlled transient elastography

For LSM by VCTE (Fibroscan 502 Touch, Echosens, Paris, France), the speed of a mechanically generated 50 Hz shear wave across the liver was measured and then converted into LSM in kilo Pascals (kPa). VCTE was performed on patients after fasting for at least 3 hours before the examination by certified physicians, nurses, or technicians who were blinded to clinical data. The M or XL probe was selected based on the automatic probe selection tool provided by the software. After patients were placed supine with the fully abducted right arm, the right liver lobe was scanned through an intercostal space to obtain a minimum of 10 valid measurements. The final results consisted of the median of all valid measurements and was considered adequate for statistical analysis if the interquartile range over the median ratio was inferior or equal to 30%.^10^

### Liver histology

The histological assessments at the local institutions were performed by hepatopathologists according to the NASH Clinical Research Network scoring system.^11^ Fibrosis stage (F0-F4) was defined as no fibrosis (F0), either mild-moderate perisinusoidal or periportal fibrosis (F1), both perisinusoidal and portal/periportal fibrosis (F2), bridging fibrosis (F3), and cirrhosis (F4).

### Outcomes

The main outcomes were the performance of Agile 3+ and 4 for the diagnosis of advanced fibrosis (≥F3) and cirrhosis (F4), respectively.

### Statistical analysis

Patient characteristics were reported as median (interquartile range) and the number of available data for numerical variables and frequency and percentage for categorical variables.

For each patient, Agile 3+ and Agile 4 were calculated as:

Considering diabetes status: yes =1, no =0 and sex: male =1, female =0,


$$\mathrm{Agile}\;4=\frac{e^{\mathrm{logit}(p_{F=4})}}{1+e^{\mathrm{logit}(p_{F=4})}}$$

with logit (*p*_F = 4_) = 7.50139−15.42498 × $\frac{1}{\sqrt{\mathrm{LSM}}}$ − 0.01378 × PLT − 1.41149 × AAR^−1^ − 0.53281 × Sex + 0.41741 × Diabetes status


$$\mathrm{Agile}\;3+=\frac{e^{\mathrm{logit}(p_{F\geq 3})}}{1+e^{\mathrm{logit}(p_{F\geq 3})}}$$

with logit (*p*_*F*≥3_) = −3.92368 + 2.29714 × ln(LSM) − 0.00902 × PLT − 0.398633 × AAR^−1^ + 1.08636 × Diabetes status − 0.38581 × Sex + 0.03018 × Age

Both scores’ performances were assessed using fibrosis stage by histology as the reference by the goodness of fit and discrimination and compared with LSM alone and FIB-4 used as predictors of advanced fibrosis and cirrhosis. The goodness of fit (the agreement between observed outcome and prediction) was evaluated using calibration plots and discrimination using the area under the receiver operating curve (AUROC).^12^ AUROC comparisons were performed using the Delong test (at a 2-sided 5% significance level).^13^ Dual cutoff approach with cutoffs of high sensitivity and high specificity already published for the different noninvasive tests was applied to rule out and rule in advanced fibrosis and cirrhosis.^8^,^9^ When appraising performance at a given cutoff, sensitivity, specificity, positive predictive value, negative predictive value, positive likelihood ratio and negative likelihood ratio were computed. Proportion of patients with indeterminate results with each noninvasive method were compared using *z*-test. Statistical analyses were computed using the R software (https://www.r-project.org/)

## RESULTS

### Baseline characteristics

**FIGURE 1:**
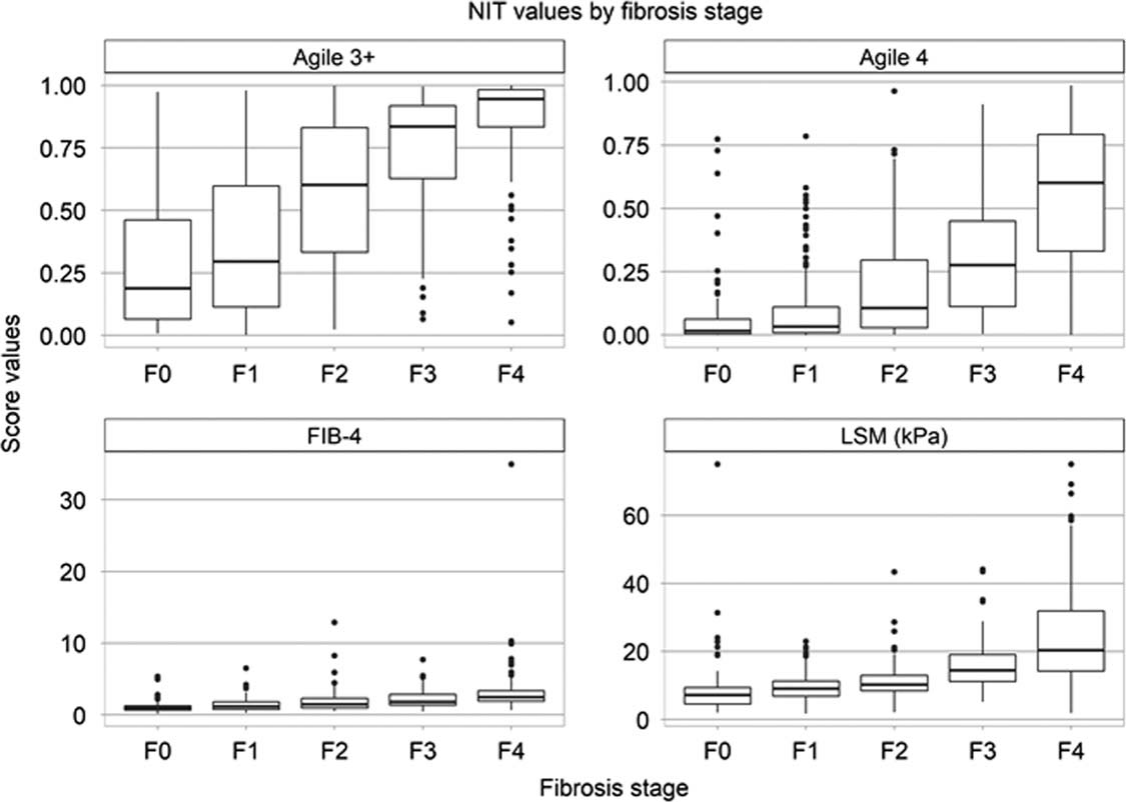
Noninvasive test values by fibrosis stage assessed by liver biopsy. Abbreviations: F0, fibrosis stage 0; F1, fibrosis stage 1; F2, fibrosis stage 2; F3, fibrosis stage 3; F4, fibrosis stage 4; FIB-4, fibrosis-4 index; LSM, liver stiffness measurement; NIT, noninvasive test.

In total, 548 of 745 subjects were included for statistical analysis (Supplemental Figure 1). Table 1 illustrates baseline characteristics of study participants overall, with obesity, and with diabetes. Median (interquartile range) age was 58 (15) years, and median body mass index was 33.3 kg/m^2^ (8.5). Fifty-three percent had diabetes, 52% had hypertension, 72% were obese, and 20% were morbidly obese. The prevalence of NASH was 50%. The median FIB-4 score was 1.67 (1.61), whereas the median LSM by VCTE was 12 kPa (10). Of these 548, 75 (14%) had F0, 114 (21%) had F1, 104 (19%) had F2, 111 (20%) had F3, and 144 (26%) had F4. The score boxplots of noninvasive values by fibrosis stage through Agile 3+, Agile 4, FIB-4, and LSM are shown in Figure 1.

**TABLE 1:** Demographic characteristics of study cohort overall, with obesity, and with diabetes

Median (IQR)	Overall	Patients with obesity	Patients with diabetes
Demographics			
Age (y)	n=548	n=359	n=292
	58 (15)	57 (15)	59 (14)
Male sex, N/n (%)	190/548 (35)	124/359 (35)	100/292 (34)
BMI (kg/m²)	n=501	n=359	n=267
	33.3 (8.5)	35.7 (7.5)	33.3 (8.1)
Comorbidities, N/n (%)			
Diabetes	292/548 (53)	199/359 (55)	292/292 (100)
Hypertension	283/547 (52)	193/359 (54)	176/292 (60)
Obesity	359/501 (72)	359/359 (100)	199/267 (75)
Morbid obesity	99/501 (20)	99/359 (28)	53/267 (20)
Blood			
AST (IU/L)	n=548	n=359	n=292
	36 (30)	40 (31)	35 (28)
ALT (IU/L)	n=548	n=359	n=292
	44 (42)	45 (46.5)	41 (39)
AST/ALT	n=548	n=359	n=292
	0.9 (0.4)	0.8 (0.4)	0.9 (0.4)
Platelets (1000/uL)	n=548	n=359	n=292
	208 (111)	214 (112)	204 (102)
HDL (mmol/L)	n=413	n=264	n=292
	1.2 (0.4)	1.2 (0.4)	1.1 (0.4)
LDL (mmol/L)	n=401	n=255	n=209
	2.5 (1.2)	2.5 (1.2)	2.4 (1.2)
Albumin (g/L)	n=534	n=353	n=287
	44 (4)	43 (4)	44 (5)
Bilirubin (µmol/L)	n=547	n=358	n=292
	10.3 (6.8)	9.3 (5.1)	10.3 (6.8)
Alkaline phosphatase (IU/L)	n=405	n=272	n=223
	81 (36)	82 (37)	81 (38)
Noninvasive tests			
FIB-4	n=548	n=359	n=292
	1.67 (1.61)	1.7 (1.5)	1.7 (1.5)
LSM by VCTE (kPa)	n=548	n=359	n=292
	12 (10)	13 (10)	12 (10)
Fibrosis, N/n (%)			
NASH	239/477 (50)	167/310 (54)	133/252 (53)
Fibrosis stage by Kleiner, N/n (%)			
F0	75/548 (14)	46/359 (13)	35/292 (12)
F1	114/548 (21)	73/359 (20)	49/292 (17)
F2	104/548 (19)	75/359 (21)	61/292 (21)
F3	111/548 (20)	70/359 (20)	72/292 (25)
F4	144/548 (26)	95/359 (27)	75/292 (26)

*Note:* Obesity is defined as BMI ≥30. Morbid obesity is defined as BMI ≥35.

Abbreviations: ALT, alanine aminotransferase; AST, aspartate aminotransferase; BMI, body mass index; F0, fibrosis stage 0; F1, fibrosis stage 1; F2, fibrosis stage 2; F3, fibrosis stage 3; F4, fibrosis stage 4; FIB-4, fibrosis-4 index; IQR, interquartile range; LSM, liver stiffness measurement; VCTE, vibration-controlled transient elastography.

### Agile 3+ in comparison with FIB-4 and LSM for the identification of advanced fibrosis

**FIGURE 2:**
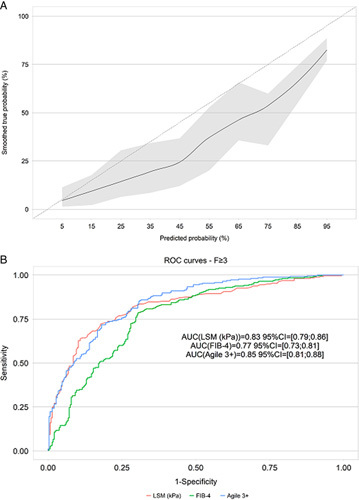
Goodness of fit of Agile 3+ (A) and ROC curves of Agile 3+, FIB-4, and LSM for the identification of advanced fibrosis (≥fibrosis stage 3) using liver biopsy as the reference. Abbreviations: FIB-4, fibrosis-4 index; LSM, liver stiffness measurement; ROC, receiver operator curve.

Figure 2A illustrates the goodness of Agile 3+. The performance of the Agile 3+ in identifying those with advanced fibrosis (≥F3) was compared with those of FIB-4 and LSM by VCTE (Table 2, Figure 2B). For the rule-out and rule-in cutoffs, we, respectively, applied the following previously published cutoffs values of <0.451 and ≥0.679 for Agile 3+, <1.3 (<65 y)/<2.0 (≥65 y) and >2.67 for FIB-4, and <8 and >9.6 for LSM.^8^,^14^,^15^

**TABLE 2:** Performance of Agile 3+ versus FIB-4 and LSM by VCTE in identifying advanced fibrosis

	FIB-4	LSM	Agile 3+
AUROC (95% CI)	0.77 (0.73; 0.81)	0.83 (0.79; 0.86)	0.85 (0.81; 0.88)
Delong test p (vs. Agile 3+)	<0.0001	0.142	NA
Rule out cutoff	<1.3 (<65 y)	<8.0	<0.451
	<2.0 (≥65 y)	—	—
Percentage of patients	45	24	34
Se	0.77 (0.718; 0.822)	0.91 (0.875; 0.945)	0.91 (0.875; 0.945)
Sp	0.64 (0.695; 0.858)	0.37 (0.315; 0.425)	0.56 (0.503; 0.617)
NPV	0.76 (0.707; 0.813)	0.83 (0.766; 0.894)	0.88 (0.834; 0.926)
LR−	0.36	0.23	0.16
Gray zone			
Percentage of patients (*p*-value vs. Agile 3+)	31 (*p*<0.001)	13 (*p*<0.001)	14
Rule in cutoff	≥2.67	>9.6	≥0.679
Percentage of patients	24	63	52
Se	0.38 (0.320; 0.440)	0.86 (0.817; 0.903)	0.80 (0.751; 0.849)
Sp	0.87 (0.831; 0.909)	0.58 (0.523; 0.637)	0.72 (0.669; 0.771)
PPV	0.72 (0.644; 0.796)	0.64 (0.589; 0.691)	0.71 (0.657; 0.763)
LR+	2.93	2.04	2.86

Advanced fibrosis defined as ≥F3.

Abbreviations: AUROC, area under the receiver operator curve; F3, fibrosis stage 3; FIB-4, fibrosis-4 index; LR+, positive likelihood ratio; LR−, negative likelihood ratio; LSM, liver stiffness measurement; NPV, negative predictive value; PPV, positive predictive value; Se, sensitivity; Sp, specificity; VCTE, vibration-controlled transient elastography.

Overall, AUROC of Agile 3+ was significantly higher than that of FIB-4 [Agile 3+: 0.85 (0.81; 0.88), FIB-4: 0.77 (0.73; 0.81), *p*<0.0001], but similar to the AUROC of LSM [0.83 (0.79; 0.86), *p*=0.142]. Percentages of patients with indeterminate results was significantly lower for Agile 3+ and LSM compared with FIB-4 (Agile 3+: 14%, LSM: 13%, FIB-4: 31%, *p*<0.0001). At the rule-out cutoff, compared with FIB-4 and LSM, Agile 3+ exhibited similar sensitivity (within 0.10) to that of LSM but better sensitivity than that of FIB-4 (Agile 3+: 0.91, LSM: 0.91, FIB-4: 0.77), similar specificity to that of FIB-4 but better specificity than that of LSM (Agile 3+: 0.56, FIB-4: 0.64, LSM: 0.37), and overall highest negative predictive value (Agile 3+: 0.88, FIB-4: 0.76, LSM: 0.83), though overall lowest negative likelihood ratio (Agile 3+: 0.16, FIB-4: 0.36, LSM: 0.23). At the rule in cutoff, Agile 3+ demonstrated similar sensitivity to that of LSM but better sensitivity than that of FIB-4 (Agile 3+: 0.80, FIB-4: 0.38, LSM: 0.86), better specificity than that of LSM but lower specificity than that of FIB-4 (Agile 3+: 0.72, FIB-4: 0.87, LSM: 0.58), similar positive predictive value (Agile 3+: 0.71, FIB-4: 0.72, LSM: 0.64), and similar positive likelihood ratio to that of FIB-4 but better positive likelihood ratio than that of LSM (Agile 3+: 2.86, FIB-4: 2.93, LSM: 2.04).

**FIGURE 3:**
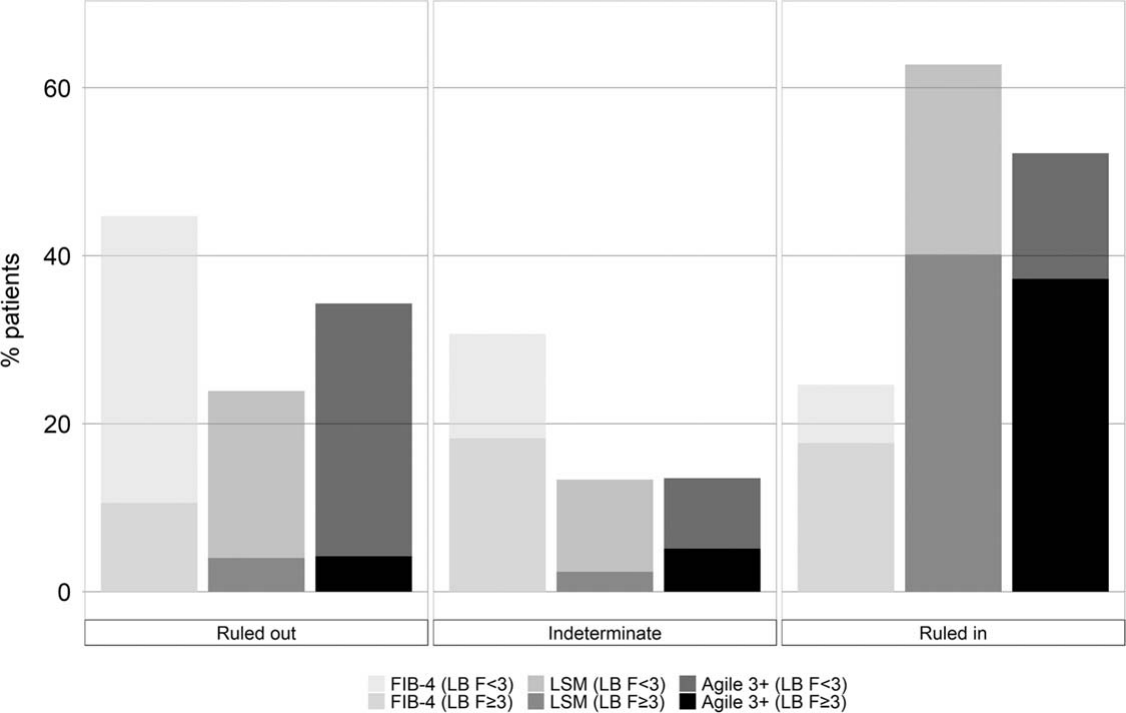
Performance of Agile 3+ versus FIB-4 and LSM by vibration-controlled transient elastography in identifying advanced fibrosis. In the rule-out zone, FIB-4 (LB F<3) represents the percentage of patients with a FIB-4 under the rule-out cutoff and with a fibrosis stage <3 (true negative). FIB-4 (LB F≥3) represents the percentage of patients with a FIB-4 under the rule-out cutoff and a fibrosis stage ≥3 (false negative). In the intermediate zone, FIB-4 (LB F<3) represents the percentage of patients with a FIB-4 between the rule-out and the rule-in cutoffs and a fibrosis stage <3. FIB-4 (LB F≥3) represents the percentage of patients with a FIB-4 between the rule-out and the rule-in cutoffs and a fibrosis stage ≥3. In the rule-in zone, FIB-4 (LB F<3) represents the percentage of patients with a FIB-4 above the rule-in cutoff and with a fibrosis stage <3 (false positive). FIB-4 (LB F≥3) represents the percentage of patients with a FIB-4 above the rule-in cutoff and a fibrosis stage ≥3 (true positive). As with FIB-4, the same interpretation applies with LSM (LB F<3), LSM (F≥3), Agile 4 (LB F<3), and Agile (LB F≥3). Advanced fibrosis is defined as ≥fibrosis stage 3. Abbreviations: FIB-4, fibrosis-4 index; LB, liver biopsy; LSM, liver stiffness measurement.

The performances of the Agile 3+, FIB-4, and LSM by VCTE in identifying advanced fibrosis (≥F3) are graphed in Figure 3. Subgroup analyses in patients with obesity and diabetes are, respectively, shown in Supplemental Tables 1 and 2 (http://links.lww.com/HC9/A122, http://links.lww.com/HC9/A123).

### Agile 4 performance in comparison to LSM for the identification of cirrhosis

**FIGURE 4:**
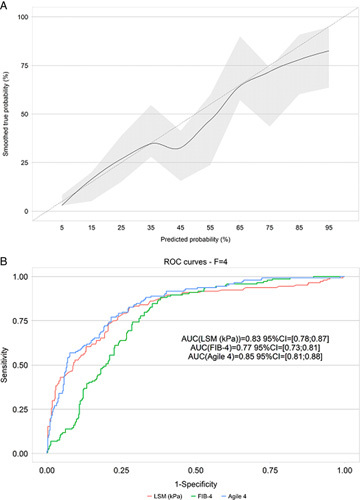
Goodness of fit of Agile 4 (A) and ROC curves of Agile 4, FIB-4, and LSM for the identification of cirrhosis (fibrosis stage 4) using liver biopsy as the reference. Abbreviations: FIB-4, fibrosis-4 index; LSM, liver stiffness measurement; ROC, receiver operator curve.

The performance of Agile 4 in identifying those with cirrhosis (F4) compared with LSM is shown in Figure 4 and Table 3. The AUROCs of Agile 4 and LSM were similar [Agile 4: 0.85 (0.81; 0.88) vs. LSM: 0.83 (0.78; 0.87), *p*=0.065]. For the rule-out and rule-in cutoffs, we, respectively, applied published cutoffs of <0.251 and ≥0.565 for Agile 4 and <8 and ≥14 for LSM.^9^,^14^ Agile 4 was not compared with FIB-4 using the dual cutoff approach because of the lack of published cutoffs for FIB-4 in identifying cirrhosis.

**TABLE 3:** Performance of Agile 4 versus LSM in identifying cirrhosis

	LSM	Agile 4
AUROC (95% CI)	0.83 (0.78; 0.87)	0.85 (0.81; 0.88)
Delong test *p* (vs. Agile 4)	0.065	NA
Rule out cutoff (Sen 90%)	<8	<0.251
Percentage of patients	24	57
Se	0.94 (0.901; 0.979)	0.83 (0.769; 0.891)
Sp	0.30 (0.255; 0.345)	0.71 (0.666; 0.754)
NPV	0.93 (0.886; 0.974)	0.92 (0.890; 0.950)
LR−	0.21	0.24
Gray zone		
Percentage of patients (*p*-value vs. Agile 4)	38 (*p*<0.001)	23
Rule in cutoff (Spec 90%)	>14	≥0.565
Percentage of patients	38	20
Se	0.77 (0.701; 0.839)	0.54 (0.459; 0.621)
Sp	0.76 (0.718; 0.802)	0.93 (0.905; 0.955)
PPV	0.54 (0.472; 0.608)	0.72 (0.635; 0.805)
LR+	3.24	7.29

Abbreviations: AUROC, area under the receiver operator curve; FIB-4, fibrosis-4 index; LR+, positive likelihood ratio; LR−, negative likelihood ratio; LSM, liver stiffness measurement; NPV, negative predictive value; PPV, positive predictive value; Se, sensitivity; Sp, specificity; VCTE, vibration-controlled transient elastography.

The percentages of patients within the indeterminate zone were significantly lower with Agile 4 (23%) versus LSM (38%) (*p*<0.0001). At the rule out cutoff, compared with LSM, Agile 4 exhibited higher specificity (Agile 4: 0.71 vs. LSM: 0.30) and negative likelihood ratio (Agile 4: 0.24 vs. LSM: 0.21) and similar sensitivity (Agile 4: 0.83 vs. LSM: 0.94) and NPV (Agile 4: 0.92 vs. LSM: 0.93). At the rule-in cutoff, Agile 4 demonstrated higher specificity (Agile 4: 0.93 vs. LSM: 0.76), positive predictive value (Agile 4: 0.72 vs. LSM: 0.52), and positive likelihood ratio (Agile 4: 7.29 vs. LSM: 3.24), but lower sensitivity (Agile 4: 0.54 vs. LSM: 0.77).

**FIGURE 5:**
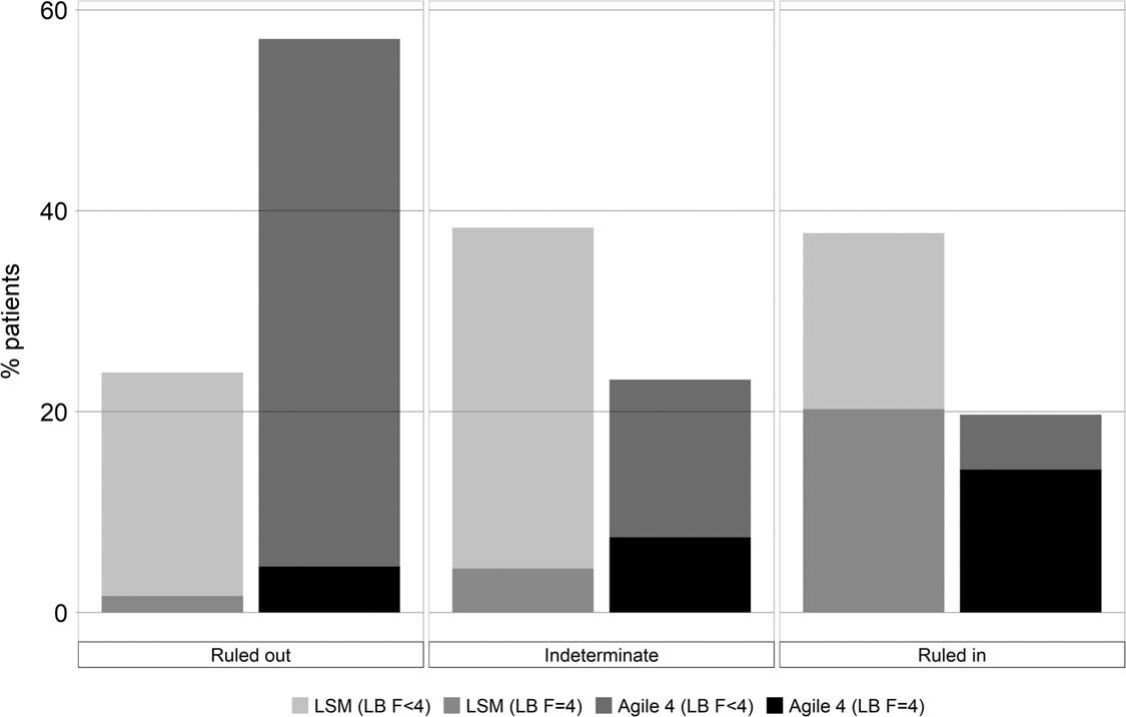
Performance of Agile 4 versus LSM by vibration-controlled transient elastography in identifying cirrhosis. In the rule-out zone, LSM (LB F<4) represents the percentage of patients with an LSM under the rule-out cutoff and with a fibrosis stage <4 (true negative). LSM (LB F=4) represents the percentage of patients with an LSM under the rule-out cutoff and a fibrosis stage = 4 (false negative). In the intermediate zone, LSM (LB F<4) represents the percentage of patients with an LSM between the rule-out and the rule-in cutoffs and a fibrosis stage <4. LSM (LB F=4) represents the percentage of patients with an LSM between the rule-out and the rule-in cutoffs and a fibrosis stage = 4. In the rule-in zone, LSM (LB F<4) represents the percentage of patients with an LSM above the rule-in cutoff and with a fibrosis stage <4 (false positive). LSM (LB F=4) represents the percentage of patients with an LSM above the rule-in cutoff and a fibrosis stage = 4 (true positive). As with LSM, the same interpretation applies with Agile 4 (LB F<4) and Agile (LB F=4). Cirrhosis is defined as fibrosis stage 4. Abbreviations: LB, liver biopsy; LSM, liver stiffness measurement.

The performances of Agile 4 and LSM by VCTE in identifying cirrhosis (F4) are graphed in Figure 5. Subgroup analyses in patients with obesity and diabetes are, respectively, shown in Supplemental Tables 3 and 4 (http://links.lww.com/HC9/A124, http://links.lww.com/HC9/A125).

## DISCUSSION

Given the clinical silence of NAFLD disease progression, it is essential to identify those with advanced fibrosis and cirrhosis, as later stages of fibrosis are associated with higher risk of mortality and necessitate therapeutic intervention.^3^ This study independently validated the Agile 3+ and 4 scores for noninvasively identifying NAFLD patients with advanced fibrosis and cirrhosis, respectively, and compared the Agile 3+ and 4 scores’ performances to those of FIB-4 and LSM.

Both the Agile 3+ and 4 scores performed superiorly to FIB-4 and LSM by VCTE and maintained good accuracy for the diagnosis of advanced fibrosis and cirrhosis, respectively. Both the Agile 3+ and 4 scores demonstrated good calibration with the curve close to the ideal calibration line. Both Agile 3+ and 4 scores exhibited good accuracy, though their AUROCs were not significantly different but numerically higher from those of LSM. The Agile 3+ and 4 scores’ strengths lie in their 2-score cutoffs approach that significantly decreased the percentages of patients in the indeterminate zone compared with FIB-4 and LSM. The Agile scores demonstrated (1) improvement in the number of patients properly ruled out with higher specificity and negative predictive value in the rule-out zone for both Agile 3+ and 4 compared with FIB-4 and LSM except for similar specificity to that of FIB-4 for Agile 3+, (2) improvement in the identification of advanced fibrosis (Agile 3+) with higher sensitivity compared with FIB-4 and higher positive likelihood ratio compared with LSM in the rule in zone, and (3) overall better discrimination with the least number of patients in the indeterminate zone compared with both FIB-4 and LSM, except for Agile 3+ (14%) compared with LSM (13%).

The improvements associated with Agile 3+ and Agile 4 are particularly important, as patients with ≥F3 and F4 are at the highest risk of developing clinical liver events and outcomes; these 2 groups were previously not sufficiently well ruled in using LSM by VCTE of FIB-4.^16^ Adding these scores will increase the confidence of users that their patients have either ≥F3 or F4 and eventually identify patients candidates for therapies more accurately in clinical practice and trigger surveillance for the occurrence of complications such as esophageal varices and HCC.

Major strengths of Agile 3+ and 4 include their noninvasive combination of serum biomarkers and LSM by VCTE and performance accuracy. Through the noninvasive identification of, respectively, advanced fibrosis and cirrhosis, the Agile 3+ and 4 scores decrease the need for invasive liver biopsies. As FIB-4 and LSM by VCTE are insufficient for ruling-in advanced stages of fibrosis, the Agile 3+ and 4 scores also reduce additional healthcare testing that might have been warranted in the case of a positive FIB-4 or LSM.^7^ Furthermore, the Agile 3+ and 4 scores’ better discrimination with reduced number of patients in the indeterminate zone will inform next steps and aid clinical decision-making. Finally, the Agile 3+ and 4 scores use dual score cutoffs that may be applied to identify advanced fibrosis and cirrhosis, respectively, in the clinical trials and clinical setting with high utility and ease.

This study has several limitations. This study is a retrospective analysis. However, the overall study cohort consisted of 4 well-characterized study populations that not only originate from multiple tertiary care centers that follow standardized criteria for performing noninvasive testing and liver biopsy but also included the entire spectrum of NAFLD. Furthermore, the study cohort was large despite its adherence to stringent study inclusion requirements including laboratory tests liver biopsy, and LSM by VCTE within 6 months. Second, the prevalence rates for advanced fibrosis (46%) and cirrhosis (26%) are higher than those in the general community, but these high prevalence rates empower robust analysis of the performances of the Agile 3+ and 4 scores in comparison to FIB-4 and LSM. Third, although histological readings were not completed centrally, readings were performed by experienced hepatopathologists at each center. Fourth, clinical uptake of the Agile 3+ and 4 scores may be limited by availability of VCTE that requires investing in training personnel and device supply. Still, VCTE remains more cost-effective and risk free than similar imaging modalities such as CT or MRI.^17^–^19^ Indeed, VCTE has already been implemented by many experts and society guidelines as an important step for assessing disease severity in NAFLD patients.^20^,^21^ In addition, the constitutive demographic data (age, sex, and presence of type 2 diabetes) and serum biomarkers (aspartate aminotransferase, alanine transaminase, and platelets) used in Agile 3+ and 4 are incorporated in the standard examination of any liver disease. The Agile 3+ and 4 scores have been included into online calculators with high clinical utility.

In summary, introducing the noninvasive Agile 3+ and 4 scores is important given its improved discrimination with decreased indeterminate rate and increased accuracy in properly ruling in and ruling out patients with late stages of fibrosis. By successfully identifying advanced fibrosis and cirrhosis, respectively, the Agile 3+ and 4 scores will aid clinicians in targeting those who are at higher risk for liver-related mortality and may benefit from therapeutic intervention and surveillance for the development of end-stage liver disease complications.
